# Scutellarin Attenuates Hypertension-Induced Expression of Brain Toll-Like Receptor 4/Nuclear Factor Kappa B

**DOI:** 10.1155/2013/432623

**Published:** 2013-09-25

**Authors:** Xingyong Chen, Xiaogeng Shi, Xu Zhang, Huixin Lei, Simei Long, Huanxing Su, Zhong Pei, Ruxun Huang

**Affiliations:** ^1^Department of Neurology, National Key Clinical Department and Key Discipline of Neurology, The First Affiliated Hospital Sun Yat-Sen University, Guangzhou 510080, China; ^2^Department of Neurology, Fujian Provincial Hospital, Fujian Medical University, Fuzhou 350001, China; ^3^Guangdong Key Laboratory for Diagnosis and Treatment of Major Neurological Diseases, The First Affiliated Hospital, Sun Yat-Sen University, Guangzhou 510080, China; ^4^Department of Neurology, The Second Affiliated Hospital, Guangzhou University of Chinese Medicine, Guangzhou 510120, China; ^5^Key Laboratory of Quality Research in Chinese Medicine, Institute of Chinese Medical Sciences, University of Macau, Taipa 999078, Macau

## Abstract

Hypertension is associated with low-grade inflammation, and Toll-like receptor 4 (TLR4) has been shown to be linked to the development and maintenance of hypertension. This study aimed to investigate the effects of scutellarin (administered by oral gavage daily for 2 weeks) on brain TLR4/nuclear factor kappa B-(NF-**κ**B-) mediated inflammation and blood pressure in renovascular hypertensive (using the 2-kidney, 2-clip method) rats. Immunofluorescence and western immunoblot analyses revealed that hypertension contributed to the activation of TLR4 and NF-**κ**B, accompanied by significantly enhanced expression of proinflammatory mediators, such as tumor necrosis factor-**α** (TNF-**α**), interleukin-1**β** (IL-1**β**), and interleukin-18 (IL-18). Furthermore, expression of the antiapoptotic protein, myeloid cell leukemia-1 (Mcl1), was decreased, and the pro-apoptotic proteins, Bax and cleavedcaspase-3 p17 were increased in combined cerebral cortical/striatal soluble lysates. Scutellarin significantly lowered blood pressure and attenuated the number of activated microglia and macrophages in brains of hypertensive rats. Furthermore, scutellarin significantly reduced the expression of TLR4, NF-**κ**B p65, TNF-**α**, IL-1**β**, IL-18, Bax and cleaved-caspase-3 p17, and increased the expression of Mcl1. Overall, these results revealed that scutellarin exhibits anti-inflammatory and anti-apoptotic properties and decreases blood pressure in hypertensive rats. Therefore, scutellarin may be a potential therapeutic agent in hypertension-associated diseases.

## 1. Introduction

Hypertension is a major risk factor for cardiovascular accidents [1] and may also accelerate the onset and progression of ischemia stroke and cerebral hemorrhage [2]. Hypertension is associated with low-grade inflammation, which may be associated with hypertension-mediated damage to target organs [3]. Inflammation in brain parenchyma can occur as a local process that can be triggered and sustained by activated glial cells, which is thought to contribute to the pathogenesis of several diseases [3]. The innate immune response, predominantly represented by Toll-like receptors (TLRs), has been shown to contribute to the development of this condition [4]. 

TLRs are first-line molecules for initiating the innate immune responses, and thus its signaling is involved in the activation of microglia by pathogens and damaged host cells. Activation of microglia by TLRs is considered to be the “classical” form of activation [5]. Activated microglia subsequently secret proinflammatory cytokines and express costimulatory molecules needed for protective immune responses to pathogens and efficient clearance of damaged tissues [6]. TLR4 is an important contributor to microglial activation and known to initiate an inflammatory cascade in response to various brain injuries [7]. TLR4 has been shown to be linked to the development and maintenance of hypertension [8]. Furthermore, TLR4 is involved in cerebrovascular diseases, including stroke [9] and neurodegeneration [10]. TLR4-mediated signaling activates the nuclear factor kappa B (NF-*κ*B) signaling pathway, which plays a critical role in immune and inflammatory responses, cell death, and survival [5, 9]. Therefore, enhanced expression of brain TLR4/NF-*κ*B may play an important role in cerebral pathology induced by hypertension.

5,6,4-Trihydroxyflavone-7-O-glucoronide (scutellarin) is a flavone and the major active component of *Erigeron* breviscapus (Vant.) Hand-Mazz, a herbal medicine in use in the Orient for the treatment of cerebrovascular diseases [11–13]. In recent years, many studies in different animals and cells models have provided evidence for the protective effects of scutellarin because of its antioxidant [12, 14, 15], antiapoptotic [14, 16–18], anti-inflammatory [19, 20], and calcium channel antagonist properties [21]. Therefore, the aim of the present study was to investigate whether scutellarin treatment reduced the expression of brain TLR4/NF-*κ*B, the inflammatory status, and blood pressure in renovascular hypertensive rats.

## 2. Materials and Methods

### 2.1. Animals

All experimental procedures were approved by the Institutional Animal Ethical Committee of Sun Yat-Sen University and were conducted according to the Guide for the Care and Use of Laboratory Animal of the National Institute of Health (Publication no. 80-23, revised 1996). A total of 24 male Sprague-Dawley rats (60–80 g) were purchased from the Center for Experimental Animals of Sun Yat-Sen University. Rats were randomly assigned into four groups (six rats per group): (1) sham-operated group (normotensive controls), (2) hypertension with normal saline (NS) treatment, (3) hypertension with low-dose (5 mg/kg per day) scutellarin, and (4) hypertension with high-dose (20 mg/kg per day) scutellarin. Scutellarin Scutellarin (Yunnan Biovalley pharmaceutical Company Ltd, Yunnan, China) was dissolved in sterile NS, and different doses of scutellarin were administered by gavage lasting for 2 weeks.

### 2.2. Hypertension Model and Drug Administration

Hypertension was induced using a 2-kidney, 2-clip method (2K2C), as described by Zeng et al. [22]. Briefly, under anesthesia with 10% chloral hydrate (3 mL/kg body weight, intraperitoneally [i.p.]), a median longitudinal incision on the abdominal skin was performed, and then the roots of both right and left renal arteries were constricted by placing ring-shaped silver clips with an inner diameter of 0.30 mm to induce hypertension. Approximately 8 weeks later, those rats with systolic blood pressure higher than 140 mmHg and without stroke symptoms were selected for the experiment. Different doses of scutellarin were administered by gavage daily for 2 weeks. In the NS group, hypertensive rats were given saline in the same volume as scutellarin. Renal arteries in sham-operated rats also underwent surgery but without placement of clips. Systolic blood pressure (SBP) was measured by an indirect tail-cuff sphygmomanometer (MRB-IIIA, Shanghai Institute of Hypertension, Shanghai, China) in conscious rats heated (heat lamp at 37°C, for 5 min) before and after renal artery constriction (at weekly intervals) for 10 weeks [23].

### 2.3. Preparation of Tissue Samples

The preparation of tissue samples was performed as described previously [24, 25]. Briefly, after 2 weeks of scutellarin treatment, rats were sacrificed under deep anesthesia with 10% chloral hydrate (5 mL/kg body weight, i.p.) and then transcardially perfused with 0.9% sodium chloride (at 4°C). The brains were removed, and the left frontal cerebral cortex and striatum were rapidly dissected and used for western immunoblotting analysis. For immunofluorescence labeling, the right frontal brain was sliced into horizontal sections (10 *μ*m thick) using the CM1900 cryostat (Leica, Heidelberg, Germany), and these section, were then fixed with 4% paraformaldehyde (in 0.01 M phosphate-buffered saline (PBS), pH 7.4).

### 2.4. Immunofluorescence Labeling

Immunofluorescence was carried out as described previously [7]. Briefly, sections were preincubated with 0.3% Triton X-100 in 0.01 M PBS (10 min), blocked with 10% normal goat serum (KPL, CA, USA) (1 h at room temperature RT), and then incubated (overnight at 4°C) with the primary antibodies (in primary antibody diluents (Dako, Denmark)): rabbit anti-TLR4 (1 : 100) (Santa Cruz Biotechnology, Santa Cruz, CA, USA), mouse anti-Neuronal Nuclei (NeuN) (1 : 400) (Chemicon, USA), mouse anti-rat glial fibrillary acid protein (GFAP) (1 : 800) (Cell Signaling Technology, Beverly, MA, USA), or mouse anticluster of differentiation 11b (CD11b or OX-42) (1 : 300) (Millipore, USA). Sections were then incubated with Alexa Fluor 555 conjugated goat anti-rabbit IgG (H + L), F(ab′)2 Fragment (1 : 1000), Alexa Fluor 555 conjugated goat anti-mouse IgG (H + L), F(ab′)2 Fragment (1 : 1000) (both from Cell Signaling Technology), or fluorescein isothiocyanate-goat anti-mouse IgG antibodies (1 : 200) (Zymed, USA) in 0.01 M PBS (1 h at RT). Sections counterstained for nuclei were exposed to 4′,6-diamidino-2-phenylindole dihydrochloride (1 : 1000) (Roche, Mannheim, Germany) and then mounted in ProLong Gold antifade reagent (P36930, Invitrogen, USA) prior to imaging. Immunoreactivity was visualized using the BX51 microscope (Olympus). Negative control sections were incubated with PBS only, and showed no positive staining (data not shown).

### 2.5. Western Immunoblot Analyses

Western immunoblotting was performed as previously described [26]. Briefly, the left frontal cerebral cortex and striatum were homogenized in lysis buffer (pH 7.6) with 0.01 *μ*g/mL phenylmethanesulfonyl fluoride centrifuged (16,400 rpm for 30 min), and then Protein concentrations were determined using a bicinchoninic acid (BCA) protein assay kit (PIERCE, USA) according to the manufacturer's instructions. Soluble protein (50 *μ*g) was separated by 4–20% gradient SDS/PAGE (sodium dodecyl sulfate polyacrylamide gel electrophoresis) (Bio-Rad) and then transferred onto polyvinylidene fluoride membranes (Millipore, USA). Membranes were blocked with 5% skim milk in Tris-buffered saline containing 0.1% Tween-20 and then incubated with the primary antibodies: rabbit anti-TLR4 (1 : 1000), rat anti-interleukin 1 beta (IL-1*β*) (1 : 1000), rabbit anti-myeloid cell leukemia-1 (mcl1) (1 : 1000) (all from Abcam, USA), mouse anti-NF-*κ*B p65 (1 : 1000) (Cell Signaling Technology, USA), goat anti-tumor necrosis factor alpha (TNF-*α*) (1 : 5000) (Novus Biologicals, USA), rabbit anti-IL-18 (1 : 300), rabbit anti-Bax (1 : 300), or mouse anti-caspase-3 p17 (1 : 300) (all from Santa Cruz Biotechnology, USA). Membranes were exposed to the secondary antibodies diluted in blocking buffer for 1 h at room temperature: horseradish peroxidase (HRP)-conjugated goat anti-mouse (1 : 6000) (EarthOx, USA), HRP-conjugated goat anti-rabbit (1 : 3000) (Cell Signaling Technology), or HRP-conjugated rabbit anti-goat IgG antibodies (1 : 3000) (Invitrogen, USA). Mouse monoclonal anti-*β*-actin (1 : 3000) (Proteintech Group Inc., USA) served as the housekeeping protein. Immunoreactive bands were detected using Chemiluminescent HRP Substrate (Millipore, USA) and visualized on Kodak X-OMAT films. The optical densities were normalized to those of *β*-actin and calculated as target protein expression/*β*-actin expression ratios (using Image J 1.42q).

### 2.6. Image Analysis and Quantification

All histological images were analyzed with Image-Pro Plus image analysis software (Media Cybernetics, Silver Spring, MD, USA) by one blinded assessor. The number of positively stained cells was counted using Image-Pro Plus image analysis software in nine comparable, nonoverlapping fields (425 *μ*m × 320 *μ*m; 3 fields per section × 3 sections per rat) and was presented as the average cell number per field on each section [27, 28].

### 2.7. Statistical Analysis

All data are expressed as the mean ± standard deviation and were analyzed by one-way analysis of variance (ANVOA) followed by the least significant difference (LSD) post hoc test. Significance was reached at values of *P* < 0.05 and *P* < 0.001. Statistical analysis was performed with Statistical Product and Service Solutions (SPSS) 13.0 (SPSS Inc., Chicago, IL, USA).

## 3. Results

### 3.1. Effect of Scutellarin Treatment on SBP

**Figure 1:**
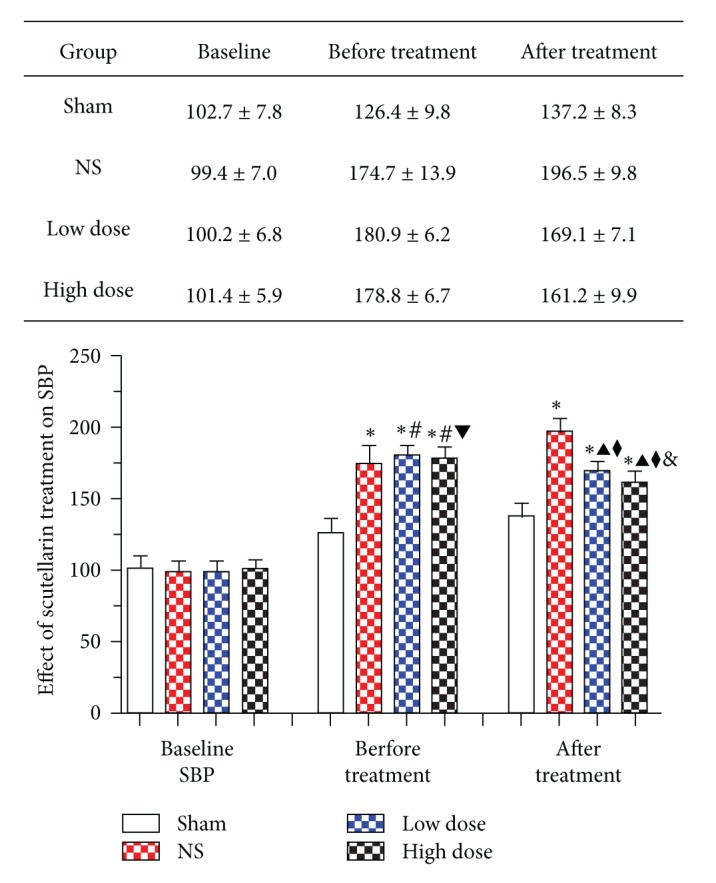
Effect of scutellarin treatment on systolic blood pressure (SBP). Baseline systolic blood pressure (SBP) (mmHg) was similar among the four experimental groups. SBP was significantly increased in hypertensive groups compared with the sham-operated group. No significant difference in SBP was found in NS rats prior to treatment. However, SBP was significantly reduced with low- and high-dose scutellarin. *P < 0.001 versus sham group; ^#^ *P* > 0.05, ^▲^ *P* < 0.001 versus NS group; ^*♦*^ *P* < 0.001, versus before treatment; ^▼^ *P* > 0.05, ^&^ *P* < 0.001 versus low-dose group.

Baseline SBP was similar between the four groups. SBP was only slightly increased in hypertension-induced rats but increased progressively to 174.7 ± 13.9, 180.9 ± 6.2, 178.8 ± 6.7, and 126.4 ± 9.8 mmHg in NS, low-dose, high-dose, and sham-operated groups, respectively (Figure 1). SBP in the NS, low-dose, and high-dose groups was significantly higher compared with the sham-operated group (*P* < 0.001). There were no incidences of stroke or death in the four groups (Table 1). No significant difference in SBP was found before treatment in NS, low-dose and high-dose groups. Compared with the NS group (196.5 ± 9.8 mmHg), scutellarin treatment significantly reduced SBP in a dose-dependent manner (*P* < 0.001). In the low-dose group, SBP was decreased by approximately 11.5 ± 6.5 mmHg, from 180.9 ± 6.2 mmHg to 169.1 ± 7.1 mmHg. In the high-dose group, SBP was reduced by approximately 17.2 ± 7.4 mmHg, from 178.8 ± 6.7 mmHg to 161.2 ± 9.9 mmHg. Furthermore, SBP was significantly different between the low-dose and high-doses group (*P* < 0.001) and was significantly higher compared with the sham-operated group (137.2 ± 8.3 mmHg) (*P* < 0.001).

### 3.2. Scutellarin Decreased the Number of Activated Microglia/Macrophages in Hypertensive Rats

**Figure 2:**
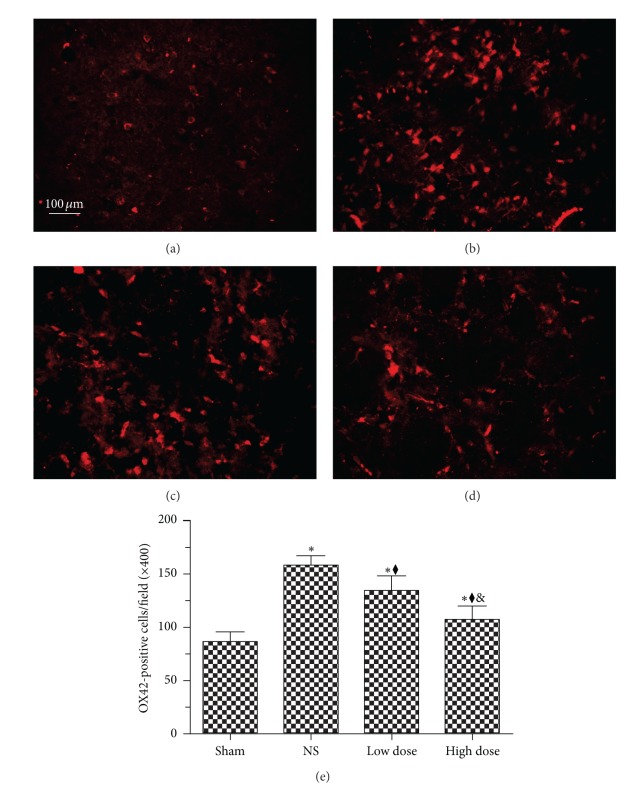
Scutellarin decreases the number of activated microglia in brain of hypertension. (a–d) Cluster of differentiation 11b (OX-42) immunostaining for microglia. (e) Quantification of OX-42-positive cells revealed that activated microglia were significantly increased in normal saline (NS) rats compared with the sham group, and treatment with scutellarin significantly reduced these numbers in a dose-dependent manner. *P < 0.001 versus sham group; ^◆^ *P* < 0.001 versus NS group; ^&^ *P* < 0.001 versus low-dose group. Scale bar = 100 **μ**m.

OX-42 labels both microglia and macrophages. Chronic hypertension induced microglia/macrophage activation (Figure 2). Compared with the sham group, the number of cells positively stained with OX-42 was significantly increased in the NS group (178.7 ± 18.5/mm^2^ versus 86.2 ± 16.8/mm^2^) (*P* < 0.001). In contrast, the number of cells positively stained with OX-42 was significantly decreased with low-dose and high-dose scutellarin, 143.1 ± 21.9/mm^2^ and 117.4 ± 17.8/mm^2^, respectively (*P* < 0.001). Furthermore, counts in the high-dose group were significantly lower compared with the low group (*P* < 0.001). 

### 3.3. Scutellarin Suppressed Hypertension-Induced Expression of Brain TLR4

**Figure 3:**
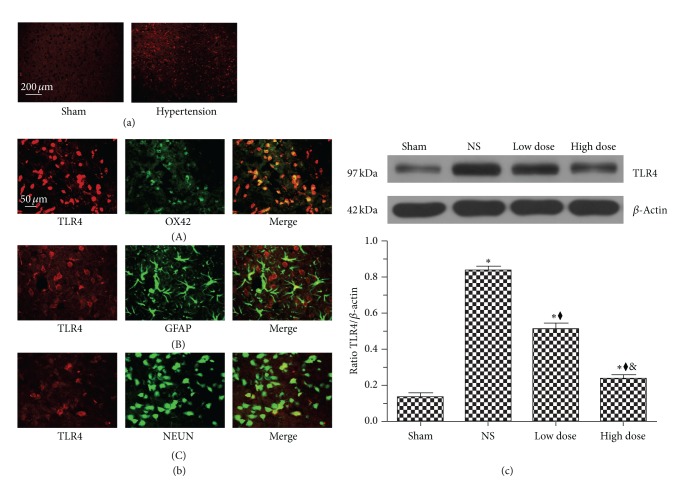
Scutellarin suppresses hypertension-induced expression of brain Toll-like receptor 4 (TLR4). (a) Chronic hypertension significantly enhanced the expression of TLR4 in the cerebral cortex. (b) Double immunofluorescence labeling indicated that the majority of TLR4 (red) was colabeled with OX-42-positive (green) cells and fewer with glial fibrillary acid protein- or Neuronal Nuclei-positive cells. (c) Western immunoblot analysis of brains of hypertensive rats showed that scutellarin dose-dependently suppressed the expression of TLR4. *P < 0.001 versus sham group; ^◆^ *P* < 0.001 versus NS group; ^&^ *P* < 0.001 versus low-dose group. (c) Scale bar = 200 *μ*m (a) and 50 *μ*m (b).

TLR4 immunoreactivity was sparse in the cerebral cortex and striatum in sham-operated rats (Figure 3(a); sham). In contrast, chronic hypertension induced higher TLR4 expression in these regions (Figure 3(a); hypertension). TLR4 was further investigated for its cellular distribution using markers for neurons (NeuN), astrocytes (GFAP) and microglia (OX-42). The majority of TLR4 (red) was colabeled with OX-42-positive cells (green) (Figure 3(b); (A)). In contrast, few TLR4-positive cells were co-labeled with GFAP-positive (green) (Figure 3(b); (B)) or NeuN-positive (green) (Figure 3(b); (C)) cells. Western immunoblot analysis showed that compared with the sham-operated group, levels of TLR4 protein were significantly increased (approximately 6-fold) in the NS group (Figure 3(c)) (*P* < 0.001). However, treatment with scutellarin significantly decreased this level in a dose-dependent manner, approximately 39.9% and 72.1% in the low-dose and high-dose groups, respectively (Figure 3(c)) (*P* < 0.001). 

### 3.4. Scutellarin Attenuated Hypertension-Induced Expression of NF-*κ*B, TNF-*α*, IL-1*β*, and IL-18

**Figure 4:**
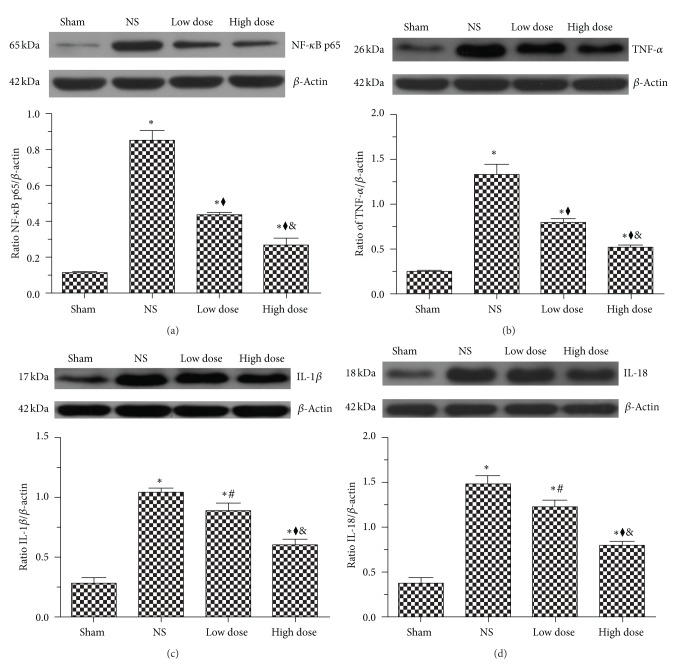
Scutellarin attenuates hypertension-induced brain expression of NF-*κ*B, TNF-*α*, IL-1*β*, and IL-18. Western immunoblot analysis for (a) NF-*κ*B p65, (b) TNF-*α*, (c) IL-1*β*, and (d) IL-18 in the rat cortex and striatum. Treatment with scutellarin significantly reduced the expression of these inflammatory markers in a dose-dependent manner. *P < 0.001 versus sham group; ^#^ *P* < 0.05, ^◆^ *P* < 0.001 versus NS group; ^&^ *P* < 0.001 versus low-dose group.

TLR4 mediates the activation of transcription factors, such as NF-*κ*B, which subsequently induces the production of inflammatory cytokines. In the present study, western immunoblot analysis showed that compared with the sham group, protein levels of NF-*κ*B p65, TNF-*α*, IL-1*β*, and IL-18 in NS rats were significantly increased by approximately 7-, 6.5-, 3.7-, and 4-fold, respectively (Figures 4(a)–4(d)) (*P* < 0.001). These proteins were significantly reduced by scutellarin, with the high-dose group inducing a markedly higher attenuation compared with the low-dose group (Figures 4(a)–4(d)) (*P* < 0.001). Levels of NF-*κ*B p65, TNF-*α*, IL-1*β*, and IL-18 were reduced to 51.1%, 61.2%, 82.9%, and 83.3%, respectively, in the low-dose group, in contrast to 31.4%, 41.9%, 57.8%, and 53.4%, respectively, in the high-dose group. Therefore, these results suggest that scutellarin reduced hypertension-mediated induction of the inflammatory response.

### 3.5. Scutellarin Treatment Upregulated the Expression of Mcl1, and Suppressed Bax and Caspase-3 p17

**Figure 5:**
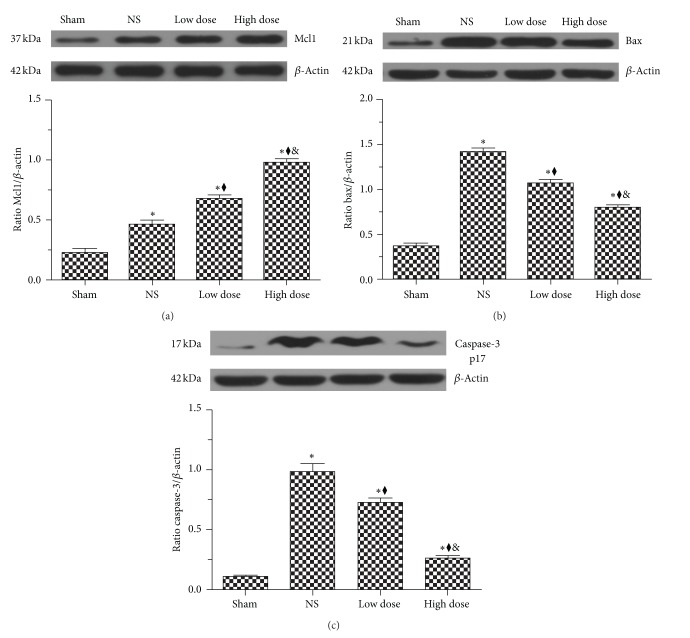
Scutellarin treatment upregulated Mcl1 and suppressed Bax, caspase-3 p17 expression. Western immunoblot analysis of protein levels of (a) Mcl1, (b) Bax, and (c) cleavedcaspase-3 p17 in the rat cortex and striatum. Scutellarin significantly upregulated Mcl1 but dose-dependently decreased Bax and cleavedcaspase-3 p17 (c). *P < 0.001 versus sham group; ^◆^ *P* < 0.001 versus NS group; ^&^ *P* < 0.001 versus low-dose group.

To investigate the potential effect of scutellarin on neuronal cell survival, we evaluated the expression of the apoptosis-related proteins, Mcl1, Bax, and cleavedcaspase-3 p17 in brains of hypertensive and sham-operated rats. Western immunoblot analysis indicated that compared with the sham group, levels of Mcl1, Bax, and cleavedcaspase-3 p17 were significantly increased in hypertensive rats (Figures 5(a)–5(c)) (*P* < 0.001). Compared with sham rats, Mcl1, Bax, and cleavedcaspase-3 p17 were significantly (*P* < 0.001) elevated in the NS group by approximately 2-, 3.8-, and 8.9-fold, respectively. Compared with the NS group, treatment with scutellarin significantly upregulated the level of Mcl1, particularly with the high-dose group (approximately 2.1-fold compared with the NS group) (Figure 5(a)) (*P* < 0.001). However, scutellarin significantly downregulated the levels of Bax and cleavedcaspase-3 p17 protein in a dose-dependent manner, to 71.4% and 73.9% (for Bax and cleavedcaspase-3 p17, resp.) for the low-dose group and 56.3% and 27.1% (for Bax and cleavedcaspase-3 p17, resp.) for the high-dose group (Figures 5(a) and 5(c)) (*P* < 0.001). 

## 4. Discussion

In the present study, we demonstrate that scutellarin is protective against chronic hypertension-induced activation of brain TLR4 and subsequent NF-*κ*B-mediated inflammatory responses. We show that scutellarin possesses anti-inflammatory and antiapoptotic properties and lowers blood pressure, thus suggesting its use as a potential therapeutic agent in hypertension-associated diseases.

In the central nervous system, TLR4 has been reported to be expressed in both microglia and astrocytes, as well as in neurons [7]. In this study, chronic hypertension augmented the expression of TLR4 predominantly in microglia/macrophage cells, indicating its involvement in chronic hypertension-induced inflammation. However, the expression of TLR4 in astrocytes and neurons also suggested their potential involvement, and thus further studies could explore this possible relationship. 

Innate and adaptive immunities have been shown to contribute to hypertension-associated end-organ damage, although the mechanism by which this occurs remains unclear [29]. Previous studies suggest that enhanced expression of TLR4 may be linked with the development and maintenance of hypertension and low-grade inflammation and augmented vascular contractility in hypertensive rats [8, 29]. Chronic hypertension causes cardiac hypertrophy, characterized by low-grade inflammation and accompanied by increased expression and activity of TLR4, and elevated gene expression of TNF-*α* and IL-6 in cardiac tissue [8]. Treatment with anti-TLR4 was shown to decrease mean arterial pressure, TLR4 protein in mesenteric resistance arteries, and serum levels of IL-6 in spontaneously hypertensive rats [29]. Furthermore, TLR4 signaling is also involved in brain damage and in neuroinflammatory processes associated with ischemic stroke and neurodegenerative diseases, such as Alzheimer's disease [10, 30–32]. Neutralizing TLR4 at the time of intracerebral hemorrhage [7] and ischemic stroke [31] provides neuroprotection. This effect may result from TLR4-mediated activation of NF-*κ*B signaling pathways linked to the transcription of many proinflammatory genes encoding for cytokines, chemokines, proteins of the complement system, and cell adhesion molecules. Findings from our study of chronically hypertensive rats revealed that in addition to reducing blood pressure, scutellarin prevented inflammatory mediated neuronal damage by suppressing microglial activation and the concomitant rise in expression of NF-*κ*B, TNF-*α*, IL-1*β*, and IL-18. The underlying mechanism involves, at least in part, inhibition of TLR4/NF-*κ*B-dependent signaling pathway. Interestingly, although treatment with scutellarin decreased SBP, the antihypertensive effect was moderate and without a dose-response relationship, suggesting that the low dose of scutellarin may have already reached the maximum antihypertensive effect. Thus, the antihypertensive action may play a minor role in the protective activity of scutellarin against hypertension-induced brain inflammation. Therefore, these results suggest that TLR4 is a promising target for the prevention and treatment of hypertension-associated diseases.

Studies in rat primary microglia and BV2 mouse microglia cell lines have shown that scutellarin inhibits LPS-induced nuclear translocation and DNA binding activity of NF-*κ*B, accompanied by reduced production of proinflammatory mediators, such as TNF-*α* and IL-1*β* [11]. Furthermore, recent reports have demonstrated the protective effects of scutellarin in the brain and heart of ischemic rats [16, 33]. In line with these results, the present study found that scutellarin decreased hypertension-mediated neuronal apoptosis, possibly resulting from reduced TLR4- and NF-*κ*B-mediated production of the proinflammatory cytokines. 

Scutellarin is a small molecule, and its neuroprotective effects have been well documented in different brain disease models [13, 33]. The present study further demonstrated its anti-inflammatory and antiapoptotic action in the hypertensive brain. However, the precise molecular mechanism by which scutellarin protects against hypertension-induced brain damage still remains elusive. Further study on the protective molecular mechanisms, pharmacokinetics and brain penetration of scutellarin will help explain its limited effects on blood pressure and provide relevant evidence for future clinical applications.

## 5. Conclusions

In summary, chronic hypertension significantly enhanced the expression of TLR4, NF-*κ*B, and the production of the proinflammatory cytokines, TNF-*α*, IL-1*β*, and IL-18 in brains of hypertensive rats. Scutellarin lowered blood pressure and provided neuroprotective effects by suppressing TLR4/NF-*κ*B-mediated inflammation. Therefore, scutellarin may have therapeutic potential against hypertension-associated diseases.

## Figures and Tables

**Table 1:** Occurrence of stroke and death in different groups of rats. There were no incidences of stroke or death in the four groups.

Group	Stroke				Death
	CH	CI	CH + CI	SAH	
Sham	0	0	0	0	0
NS	0	0	0	0	0
Low dose	0	0	0	0	0
High dose	0	0	0	0	0

CH: cerebral hemorrhage; CI: cerebral infarction; SAH: subarachnoid hemorrhage.
